# Supplementary material for: Stream-specific feedback inputs to the primate primary visual cortex
Source: Nat Commun. 2021 Jan 11;12:228. doi: 10.1038/s41467-020-20505-5 (PMC7801467; doi:10.1038/s41467-020-20505-5)
Supplement: Supplementary file 1 — Supplementary Figure [file 41467_2020_20505_MOESM1_ESM.pdf]

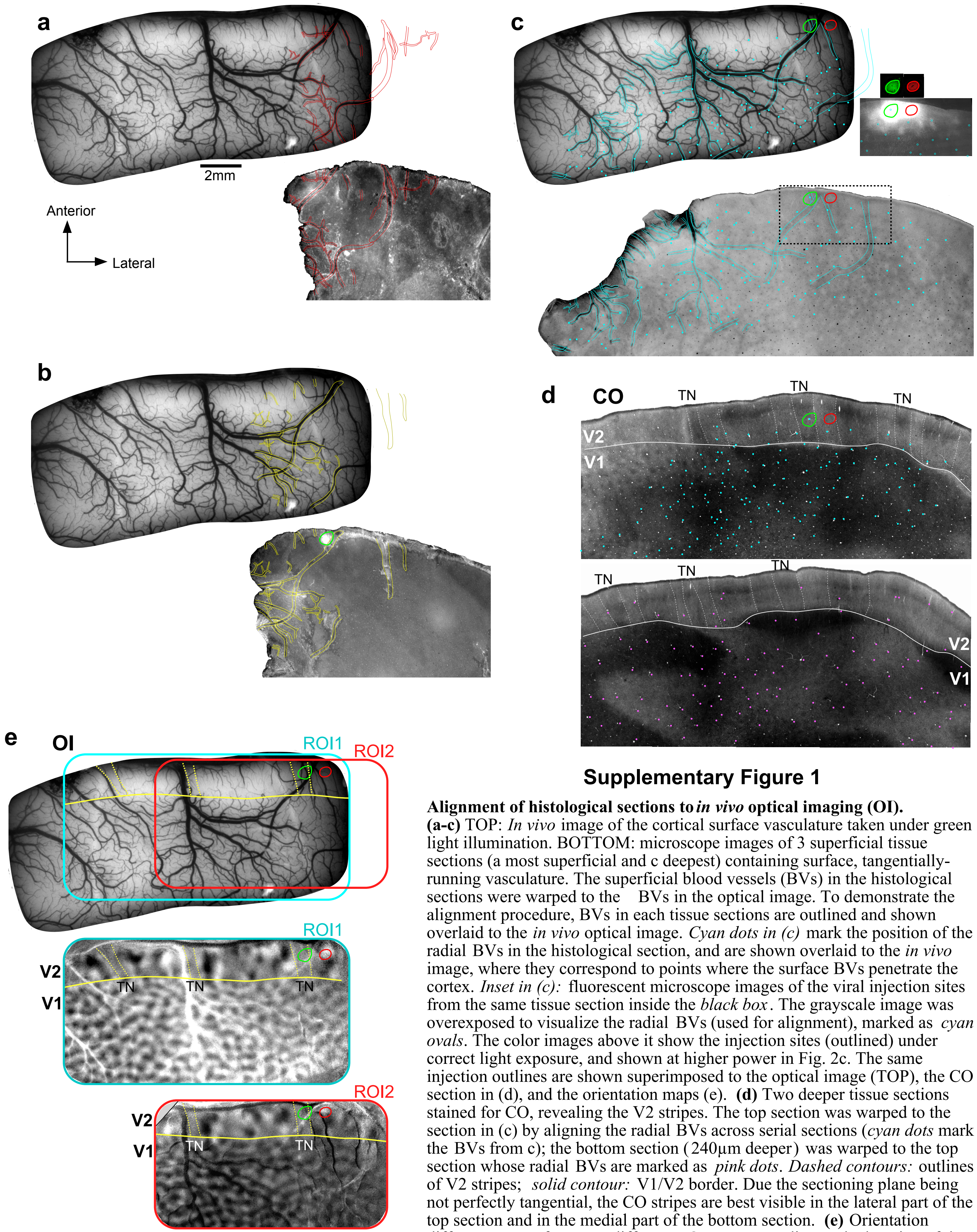

Supplementary Figure 1

Alignment of histological sections to *in vivo* optical imaging (OI).
(a-c) TOP: *In vivo* image of the cortical surface vasculature taken under green light illumination. BOTTOM: microscope images of 3 superficial tissue sections (a most superficial and c deepest) containing surface, tangentially-running vasculature. The superficial blood vessels (BVs) in the histological sections were warped to the BVs in the optical image. To demonstrate the alignment procedure, BVs in each tissue sections are outlined and shown overlaid to the *in vivo* optical image. *Cyan dots in (c)* mark the position of the radial BVs in the histological section, and are shown overlaid to the *in vivo* image, where they correspond to points where the surface BVs penetrate the cortex. *Inset in (c):* fluorescent microscope images of the viral injection sites from the same tissue section inside the *black box*. The grayscale image was overexposed to visualize the radial BVs (used for alignment), marked as *cyan ovals*. The color images above it show the injection sites (outlined) under correct light exposure, and shown at higher power in Fig. 2c. The same injection outlines are shown superimposed to the optical image (TOP), the CO section in (d), and the orientation maps (e). **(d)** Two deeper tissue sections stained for CO, revealing the V2 stripes. The top section was warped to the section in (c) by aligning the radial BVs across serial sections (*cyan dots* mark the BVs from c); the bottom section (240µm deeper) was warped to the top section whose radial BVs are marked as *pink dots*. *Dashed contours:* outlines of V2 stripes; *solid contour:* V1/V2 border. Due the sectioning plane being not perfectly tangential, the CO stripes are best visible in the lateral part of the top section and in the medial part of the bottom section. **(e)** Orientation difference maps from two different ROIs corresponding to the location of the *cyan and red boxes* at the top. *Yellow contours* outline the *TN* stripes and the V1/V2 border as revealed in the orientation maps.

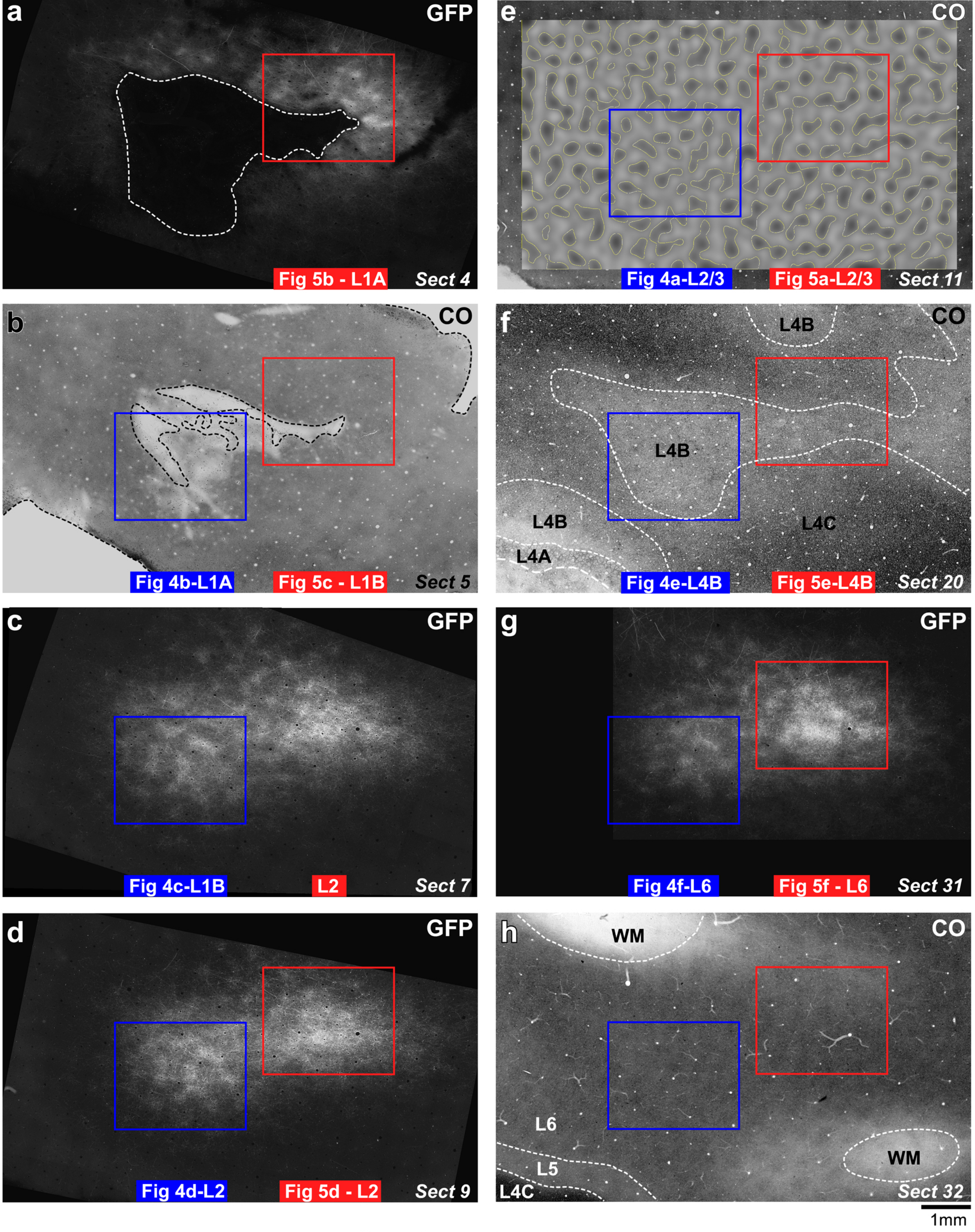

Supplementary Figure 2

Case MK356RH. Low power view, and laminar location, of the two terminal fields of FB connections arising from the thick (Left field) and thin (Right field) stripe injections shown in Fig. 3. *Blue* and *red boxes:* locations of the terminal FB field regions analyzed for the thick and thin stripe injection, respectively, and corresponding to the images shown in Figs. 4-5, as indicated below each respective box. To demonstrate the laminar location of the labeled terminal FB fields, we show CO-staining of the same section used for the GFP-label analysis (b) or of immediately adjacent sections (e,f,h). Sections in (a-h) are aligned and presented in sequence from the most superficial (a) to the deepest section (section # indicated at the bottom right corner in each panel). The section in (f) is immediately deeper to the section in Figs. 4e,5e. Scale bar under (h) valid for all panels.

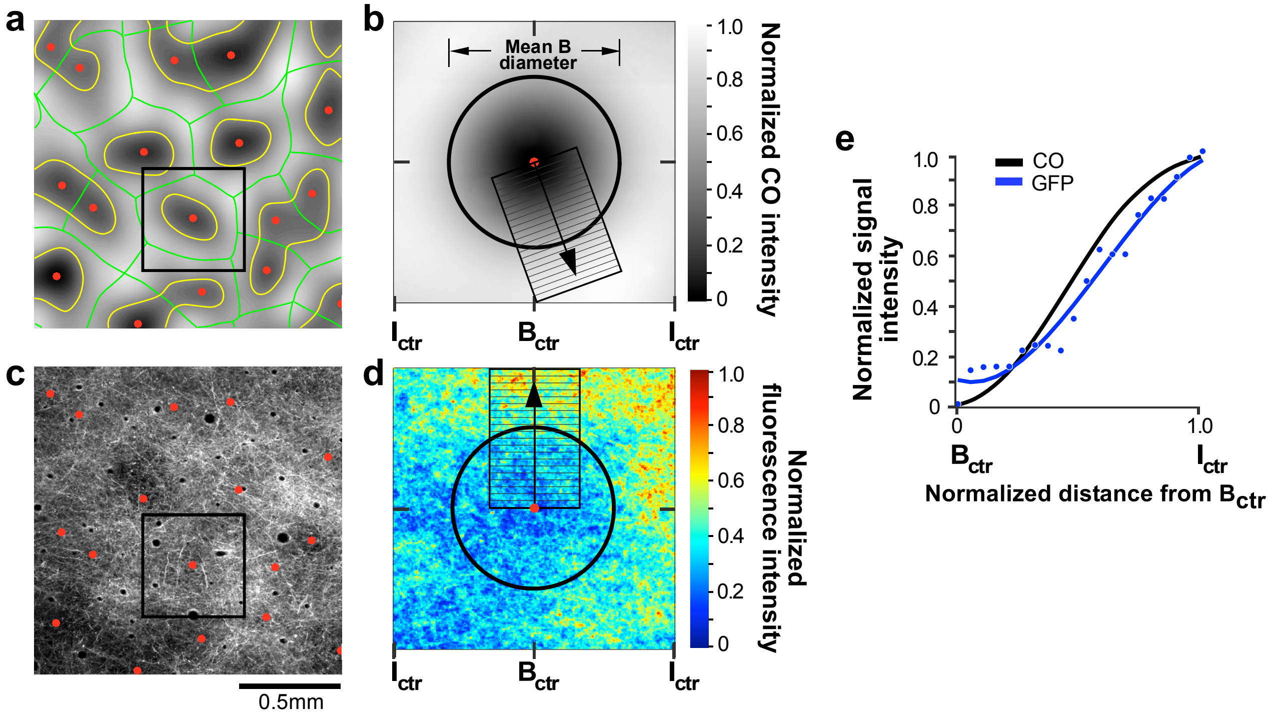

Supplementary Figure 3

Quantitative analysis of the distribution of FB axons across V1 CO compartments.
(a) Filtered CO stained section through V1 L2/3. *Yellow and green contours and red dots:* blob borders, interblob and blob centers, respectively, extracted as described in the Methods. All blobs and interblobs in this map are in vertical register with the V1 FB-axon label in (c), and were used to compute the heat map in (d). B*lack box:* ROI representing the average blob+interblob size, computed from the CO map encompassing the full extent of the labeled FB fields across layers. This ROI was centered on each blob and used to compute fluorescent signal intensity in (d). **(b)** Heat map of CO intensity, obtained by summing CO intensity across all blob ROIs for the case, and normalizing to maximum intensity. *Black circle*: mean blob diameter for all blobs used to compute the heat map. *Black box:* a 200 µm-wide window, centered on the region of highest CO intensity in the heat map, was divided into 20 bins, from the blob center to the interblob center. The mean signal intensity in each bin was normalized to the highest intensity value across all bins and plotted as a function of distance from the blob center (B_{ctr}) in panel (e) (*black curve*). **(c)** Fluorescent microscope image of GFP-labeled FB axons in a section through V1 L2/3, aligned to the section in (a). *Red dots* mark the blob centers from (a). The fluorescent signal intensity was measured in this section within the ROI from (a) centered on each blob overlaying the FB label in the layer. **(d)** Heat map of fluorescent signal intensity obtained by summing fluorescent intensity across all blob ROIs in (c), and normalizing to maximum intensity. *Black circle:* mean blob diameter from (b). The *black box* is the region of highest fluorescence intensity in the heat map used to generate the plot in panel (e) (*blue curve*). **(e)** Plots of normalized CO and fluorescent signal intensities as a function of distance from the B_{ctr}, measured within the black boxes shown in (b) and (d), respectively. Scale bar valid for (a,c).
